# A Lecture on Hematemesis; or Vomiting of Blood

**Published:** 1839-03-09

**Authors:** N. Chapman

**Affiliations:** Professor of the Theory and Practice of Physic, in the University of Pennsylvania


### MEDICAL EXAMINER.

DEVOTED TO MEDICINE, SURGERY, AND THE COLLATERAL SCIENCES.

No. 10.] PHILADELPHIA, SATURDAY, MARCH 9, 1839. [Vol. II.

### A LECTURE ON HEMATEMESIS; OR VOMITING OF BLOOD.

*By* N. Chapman, M. D., *Professor of the Theory and Practice of Physic, in the University of Pennsylvania.*

[Reported for this Journal.]

For a long time it was supposed that the discharge here came uniformly from the stomach. But it being ascertained that similar extravasations do also take place from the intestines, the liver, and spleen, and are occasionally ejected by puking, the whole of these cases have been comprehended under the same head by Pinel, Good, and some other of the modern writers. Even thus extended in its meaning, the term is still a very bad one, expressive only of a symptom, and of that, imperfectly.

An attack of hematemesis sometimes comes on without any premonition, the ejection of blood being the very first occurrence. But, oftener, it is preceded by the ordinary signs of vomiting. Nor is it unusual for these phenomena to preexist, which belong to the condition vaguely denominated dyspepsia. Except, indeed, in the most acute seizures, we shall find anorexia, or the reverse, a voracious or an irregular appetite, oppression after eating, sometimes tenderness of the epigastrium, and furred tongue, in the centre and at the root, with florid edges and tip, and constipated bowels. Cases more inveterate, are marked, also, by cardialgia, flatulence, sour, fœtid eructations, palpitations of the heart, dry skin, pale, or sallow and doughy, depression of spirits, muscular weakness, and a feeble or small corded and accelerated pulse. Mostly, under all circumstances, the attack is anticipated by alternate chilliness and flushes, a sense of tension of the stomach, and by burning or pricking in it, with weight and anxiety about the præcordia, nausea and confusion of the senses, with a disposition to syncope, attended by coldness of surface, and a still weaker pulse, increased debility, and much jactitation.

Not a few of these latter symptoms, however, are referrible to oppression of the stomach from a mass of blood in it, collected previously to vomiting, and it may be remarked in confirmation of the conjecture, that on its being thrown up, the greatest relief is for a time afforded. But unless the hæmorrhage is checked, the same train of affections recurs, till finally, absolute exhaustion takes place. The ejected blood, varies, as well in quantity as quality,—small, or extremely copious, dark and clotty, or florid and fluid—the latter not common—sometimes resembling tar in colour and consistence, and in other instances, like coffee grounds, or the sediment of port wine.

As suggested however, the effusion in hematemesis, may proceed from other of the abdominal viscera, or be vicarious of the suppression of the sanguineous discharge of some remoter or less connected organ. The upper portion of the intestinal tube being concerned, the symptoms are nearly the same as in gastric hæmorrhage, and issuing from the lower bowels; when not the product of typhoid dysentery, there is a sense of weight and oppression, very characteristic, in the hypogastric and pelvic regions.

Emanating from the liver, it is entitled melæna or morbus niger. Hippocrates considered the fluid as consisting of black bile or grumous blood, and the same notion was long entertained. The modern authorities, however, restrict these terms, to hæmorrhage only, including under them, such as proceeds from any of the abdominal contents, by the mouth or anus, provided the fluid be dark.

Commonly, when the liver or spleen is the seat of the effusion, with a loaded feeling, sometimes, an obvious distension in the right or left hypochondrium is observable, according as the one or the other organ may be implicated, accompanied, in some instances, by vomiting or purging, headache, sallowness of complexion, particularly if the liver be concerned, some fever or entire absence of it, a low and feeble pulse, a heated or cold surface, and occasionally edema, of the face or of the lower extremities, or ascites, or both. Yet, I have known such hæmorrhages, independently of any apparent disorder or vitiation of system, breaking out unexpectedly, owing perhaps to sudden engorgements of the portal circulation. As to the phenomena of the vicarious discharges, they require no special recital. An attack of these is usually sudden, indicative of an afflux of blood to the stomach, productive of the symptoms of the primary affection of that viscus, with, at the same time, a suppression of the original discharge.

From which ever of the preceding sources it may come, the quantity of blood evacuated, is on some occasions enormous. Cases of ordinary gastric hæmorrhage frequently occur where several pints are voided, and I shall hereafter mention others connected with ulceration of the stomach, in which the amount was gallons in a few days. These latter however, are perhaps, not vital hæmorrhages.

Nor may it be less from the other structures. Examples are recorded of immense discharges of blood under such circumstances, one of the most remarkable of which, is that by Michelotti in the Transactions of the Royal Society, for 1731, where a young man with enlargement of spleen, threw up in two hours more than twelve pounds of blood, and finally recovered.

In 1813, I was called to a man from the country, for supposed dropsy, whose abdomen was immensely distended, and his lower limbs edematous, with general cachexy. The case being equivocal, Dr. Hewson was brought into consultation, and very soon after we saw him an evacuation took place upwards and downwards, particularly from the bowels, at first so copious and incessant, that a succession of chamber pots was filled. The evacuation continued for two weeks, though not as largely, till finally about eight gallons were voided. The abdominal intumescence progressively subsided, and in the course of a month he returned home apparently doing well. Whether the liver or spleen was concerned in this case, could not be accurately determined, though the latter was suspected.

Nearly about the same time, I had under my care a mariner lately from India, whose case presented very much the same appearance as the preceding, with, however, more unequivocal evidence of hepatic affection. During my attendance on him, he was suddenly seized with vomiting of black dissolved blood, which scarcely intermitted for three days, when he expired, having previously thrown up eight quarts, as nearly as could be ascertained.

Not long afterwards I attended a case in the Almshouse Infirmary, of chronic hepatitis, in which the discharge of the same sort of blood, chiefly from the bowels, averaged a pint daily, for more than a fortnight, and which ultimately recovered.

During the summer of 1829, I visited, with Dr. Rhea Barton, an aged gentleman having jaundice, who, in twenty-four hours, evacuated, probably, three or four gallons of this grumous fluid. He expired with it flowing from his bowels;—and, subsequently, I saw, with Drs. Parrish and Sharpless, a young man, who, apparently in good health, was suddenly, and without any premonition, seized, while walking in the street, with the same sort of discharge, where the quantity could not have been less, in half the period. Nothing which we attempted was of any avail, and he sank completely exsanguineous. It seemed highly probable that the hæmorrhage came from the liver.

In 1835, I had a case, with Dr. Morton, very analogous to the foregoing one, in a young lady, who, in previous good health, was awakened out of her sleep by a purging of blood so profuse, that, on my arrival, I found the bed filled with it. Continuing in this way for the greater part of the night, till a prodigious, though uncertain quantity escaped, it gradually ceased the next day, and she recovered. There was here every manifestation of intestinal hæmorrhage.

Lately, I attended, with Dr. Pancoast, a distinguished member of the bar of this city, who, for some time previously having laboured under some slight symptoms of dyspepsia, was, without any direct premonition, attacked with vomiting and purging of dark, grumous blood, so copious, that in despite of our efforts, he died from exhaustion on the third day, though the hæmorrhage was early checked.

Further instances might be adduced to illustrate the extent, the danger, and even fatality, of this hæmorrhage. To account for such immense losses of blood is difficult,—and were they not so well attested, would be incredible. Yet they are not wholly inexplicable. We are aware of the copiousness of the effusion sometimes, from very limited external surfaces, of which we have ocular proof,—and it is not improbable, that in the visceral cases, by chronic congestion of an atonic nature, an enormous amount of blood had previously accumulated in the affected organ in a stagnant state, ultimately poured into the alimentary canal. Every practitioner of experience has seen the whole abdomen distended by such a condition of the liver and spleen especially,—and there is an instance reported of the latter viscus having weighed ninety-three pounds, and many of prodigious dimensions. An escape of blood under these circumstances, is very different in its effects from the loss of it directly out of the circulation, and resembles more the exhaustion induced by the sudden abstraction of the extravasated fluid in ascites.

(To be continued.)

